# K2 Transfection System Boosts the Adenoviral Transduction of Murine Mesenchymal Stromal Cells

**DOI:** 10.3390/ijms22020598

**Published:** 2021-01-09

**Authors:** Madalina Dumitrescu, Ana Maria Vacaru, Violeta Georgeta Trusca, Ioana Madalina Fenyo, Radu Ionita, Anca Violeta Gafencu

**Affiliations:** Institute of Cellular Biology and Pathology “N. Simionescu”, 8, B.P. Hasdeu Street, 050568 Bucharest, Romania; madalina.dumitrescu@icbp.ro (M.D.); ana.vacaru@icbp.ro (A.M.V.); violeta.trusca@icbp.ro (V.G.T.); madalina.fenyo@icbp.ro (I.M.F.); radu.ionita@icbp.ro (R.I.)

**Keywords:** mesenchymal stromal cells, NOD-derived MSC, C57BL/6-derived MSC, BALC/c-derived MSC, adenovirus, K2 transfection system, transduction

## Abstract

Adenoviral vectors are important vehicles for delivering therapeutic genes into mammalian cells. However, the yield of the adenoviral transduction of murine mesenchymal stromal cells (MSC) is low. Here, we aimed to improve the adenoviral transduction efficiency of bone marrow-derived MSC. Our data showed that among all the potential transduction boosters that we tested, the K2 Transfection System (K2TS) greatly increased the transduction efficiency. After optimization of both K2TS components, the yield of the adenoviral transduction increased from 18% to 96% for non-obese diabetic (NOD)-derived MSC, from 30% to 86% for C57BL/6-derived MSC, and from 0.6% to 63% for BALB/c-derived MSC, when 250 transduction units/cell were used. We found that MSC derived from these mouse strains expressed different levels of the coxsackievirus and adenovirus receptors (MSC from C57BL/6≥NOD>>>BALB/c). K2TS did not increase the level of the receptor expression, but desensitized the cells to foreign DNA and facilitated the virus entry into the cell. The expression of Stem cells antigen-1 (Sca-1) and 5′-nucleotidase (CD73) MSC markers, the adipogenic and osteogenic differentiation potential, and the immunosuppressive capacity were preserved after the adenoviral transduction of MSC in the presence of the K2TS. In conclusion, K2TS significantly enhanced the adenoviral transduction of MSC, without interfering with their main characteristics and properties.

## 1. Introduction

Mesenchymal stromal cells (MSC) are multipotent cells, that can differentiate into adipogenic, chondrogenic, and osteogenic lineage [1]. Facile isolation, plastic adherence, and the availability of multiple sources of extraction, such as the bone marrow [2], the vascular fraction of the adipose tissue [3,4], the placenta [5], and the umbilical cord [6], empowered the exploitation of MSC for cell-based therapy and transplantation. MSC have been characterized in humans and mice based on a series of positive cellular markers such as Stem cells antigen-1 (Sca-1), 5′-nucleotidase (CD73), Thy-1 (CD90), hyaluronate receptor (CD44), and endoglin (CD105) [7]. MSC represent an excellent material for regenerative medicine due to their pluripotency, their ability to differentiate into various cell types, and their immunomodulatory functions [8]. Since MSC do not possess co-stimulatory molecules for complete T cells activation, they are immune-privileged cells that do not elicit an immune response of the host in the context of allogeneic transplant [9]. MSC display strong immunomodulatory properties by suppressing the proliferation and function of lymphocytes and natural killer cells [10]. These unique features make MSC safe and promising tools for the treatment of immune-mediated disorders (such as graft-versus-host disease) and autoimmune diseases (such as type I diabetes, inflammatory bowel disease), as well as in regenerative medicine [11,12,13].

MSC represent great vehicles for gene therapy due to their potential to migrate towards diseased tissues such as tumors [14], the atherosclerotic plaque [15], or other lesioned tissues such as the ischemic myocardium [16]. In various experimental therapies, MSC were used as naïve and/or engineered cells [8,17]. Usually, a transgene is inserted in the MSC genome by lentiviral or retroviral transduction, performed with relatively high efficiency, but after insertion in the genome, it is continuously overexpressed. In contrast, the adenoviral transduction has the advantage of epigenomic expression of the transgene, which does not affect the genome permanently, as well as the ability to transduce low proliferating and quiescent cells, and the capacity to accommodate large pieces of exogenous DNA. Moreover, adenoviral transduction induces a high level of expression for a determined period, with relatively low toxicity [18]. This may be of high value in many cases in which the prolonged-expression of a gene is not desired. For these reasons, adenoviral vectors were the first viral-based vectors used for developing human gene therapies [19].

The adenovirus entry into the host cell is facilitated through a receptor-mediated process. The knob domain of the viral fiber capsid protein attaches to the coxsackie and adenovirus receptor (CAR) expressed on the host cell membrane [20], followed by the interaction between the capsid penton protein and αVβ 3 or αVβ 5 integrins present on the target cells and the internalization of the viral particle by the cell [21]. The major limitation of adenoviral gene transfer is the poor transduction efficiency of the cells that lack CAR receptors or integrins such as hematopoietic or mesenchymal stromal cells [22,23]. Thus, non-viral compounds (such as the polyamine-based transfection reagent named GeneJammer, poly-l-lysine, and polyethyleneimine) in complex with adenovirus were reported as very effective in facilitating the adenoviral binding to MSC and, thus, increasing the transduction efficiency [24,25,26]. Many researchers reported the increase in the adenoviral transduction effectiveness of other cells resistant to gene transfer by using various cationic lipids or polymer compounds: polybrene increased the adenovirus infection efficiency of immortalized mouse embryonic fibroblasts [27], Lipofectamine increased the efficacy of adenovirus-mediated gene transfer into primitive hematopoietic cells [28], and cholesterol enhanced the human fibroblasts transduction yield [29]. Bosnjak and collaborators [30] reported the K2 Transfection System (K2TS from Biontex Laboratories GmbH, München, Germany) as a very potent booster of the electrotransfection efficiency of the B16F10 melanoma cell line. K2TR has two compounds, namely K2 Multiplier (K2M), which blocks factors involved in the signaling cascade after foreign DNA detection, and K2 Transfection Reagent (K2TR), which is a powerful cationic lipid favoring DNA entry.

Here, we showed that from all the potential adenoviral transduction strategies tested, K2 Transfection System boosted the adenoviral transduction of MSC. We optimized the concentration of the K2TS components to obtain a maximum transfection yield of MSC derived from three mouse strains: non-obese diabetic (NOD), C57BL/6, and BALB/c. We demonstrated that the transduction of MSC in the presence of K2TS did not affect the key features of NOD-MSC such as the expression of the specific surface markers, differentiation capacity, and immunomodulatory properties.

## 2. Results

### 2.1. Adenoviral Transduction of MSC and the Effect of Potential Boosters

#### 2.1.1. Adenoviral Transduction of MSC Isolated from NOD, C57BL/6, and BALB/c Mice

The adenoviral system AdEasy developed by B. Vogelstein [31] was used to pack an adenovirus carrying cDNA encoding GFP protein (AdV) as we described before [32].

**Figure 1:**
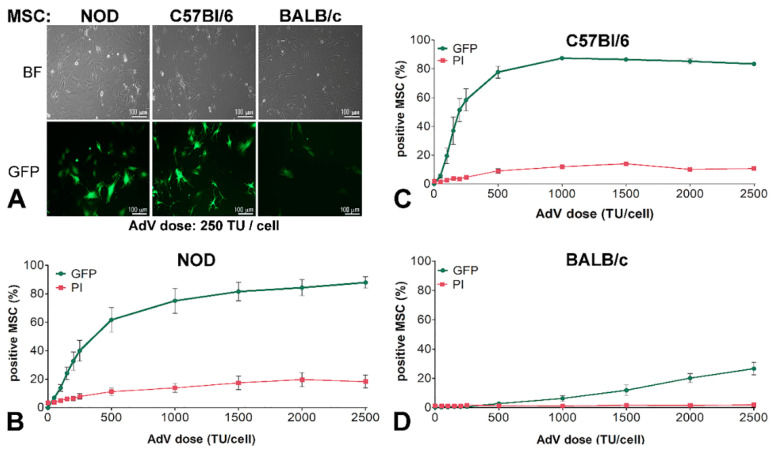
Adenoviral transduction efficiency of murine mesenchymal stromal cells (MSC) isolated from three mouse strains. (**A**) Microscopy of non-obese diabetic NOD-MSC, C57BL/6-MSC, and BALB/c-MSC, transduced with 250 transduction units (TU)/cell adenovirus for GFP expression: BF—Bright field and GFP—fluorescence microscopy. (**B**–**D**) Dose-dependent transduction of MSC derived from NOD, C57BL/6, and BALB/c strains. The cells were incubated with increasing doses of adenovirus ranging from 0–2500 TU/cell; after 48 h the GFP expressing cells and the cell death determined by propidium iodide (PI) incorporation were evaluated by flow cytometry. The dose-dependent curves are different for NOD-MSC (**B**), C57BL/6-MSC (**C**), and BALB/c-MSC (**D**).

MSC isolated from the bone marrow of NOD (NOD-MSC), C57BL/6 (C57BL/6-MSC), and BALB/c (BALB/c-MSC) mouse strains were transduced with 250 transduction units/cell (TU/cell) AdV. As shown in Figure 1A, the cells derived from these three strains were transduced with different yields, and the GFP expression was higher in C57BL/6-MSC (~41%) and NOD-MSC (~22%) than in BALB/c-MSC (~1%), as observed by fluorescence microscopy. Then, we tested the adenoviral dose-dependent GFP expression in these cells. The MSC were transduced with an increasing dose of AdV ranging from 50 to 2500 TU/cell and, 48 h after transduction, the GFP expression in each cell line was evaluated by flow cytometry. Based on this analysis, we plotted the dose-dependent curves which revealed a different shape for NOD-, C57BL/6-, and BALB/c-MSC, respectively (Figure 1B–D, green lines). NOD- and C57BL/6-MSC presented a linear dependence at adenoviral doses lower than 250 TU/cell, but the slope for NOD-MSC (0.164 ± 0.02) is smaller as compared with that obtained for C57BL/6-MSC (0.255 ± 0.03), as shown in Figure 1B,C. The transduction curve of C57BL/6-MSC reached a maximum of ~87% for 1000 TU/mL, while for NOD-MSC the transfection yield increased till the maximum dose tested (2500 TU/cell) when the yield reached 87%.

The cell death induced by the adenovirus was dose-dependent, reaching a maximum of ~20% at 2500 TU/cell in the case of NOD-MSC (Figure 1B, red line). For C57BL/6-MSC the cell death was increasing up to a dose of 250 TU/cell, and then the curve was flattened at ~14% as shown in Figure 1C, red line.

By contrast, for BALB/c-MSC, the adenoviral transduction was not efficient at doses lower than 1000 TU/cell, after which the yield increased linearly till 2500 TU/cell when 27% of the cells expressed GFP (Figure 1D, green line). The viability of BALB/c-MSC was not affected by the adenovirus, not even at the higher doses, for which the cell death was only 2% (Figure 1D, red line).

#### 2.1.2. Adenoviral Transduction of MSC in the Presence of Different Potential Enhancers of the Transduction

**Figure 2:**
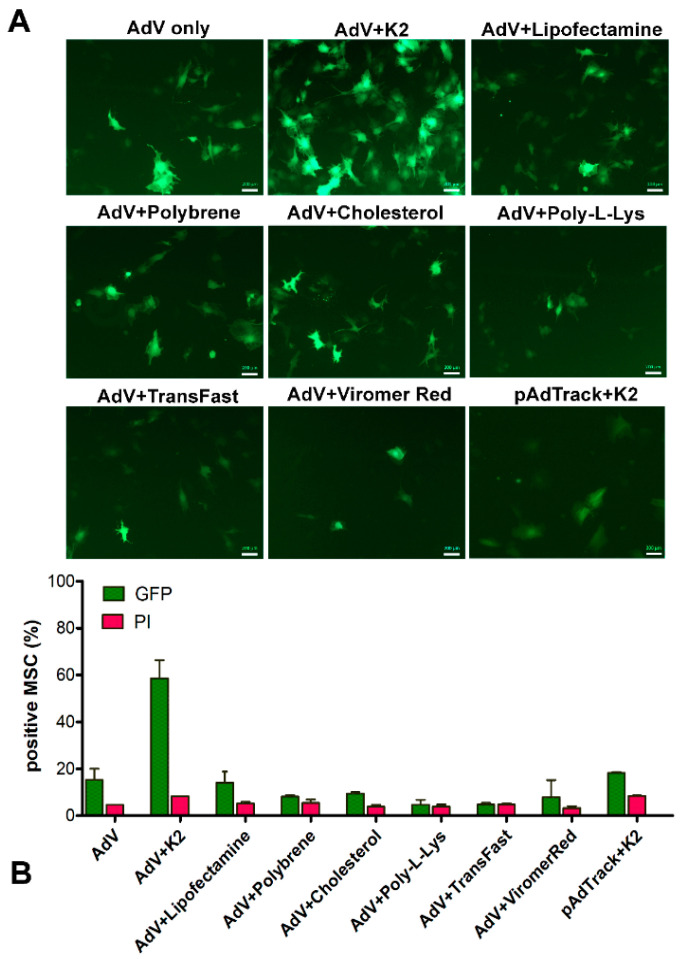
Adenoviral transduction of murine mesenchymal stromal cells in the presence of different potential transduction boosters. To induce GFP expression, MSC were incubated with 250 TU/cell adenovirus alone (AdV only) or in the presence of the K2 Transfection System (K2TS) (AdV + K2), Lipofectamine 3000 (AdV + Lipofectamine), 10 μg/mL Polybrene (AdV + Polybrene), 2 μg/mL free cholesterol (AdV + Cholesterol), 1 μg/mL poly-L-Lysine (AdV + Poly-L-Lys), TransFast (AdV + TransFast), or Viromer Red (AdV + ViromerRed). In parallel, MSC were transfected with pAdTrack-CMV using K2TS (pAdTrack + K2). After 48 h, the GFP expression was observed by fluorescence microscopy (**A**), and the number of the GFP-expressing cells was determined by flow cytometry (**B**). The percentage of GFP-positive cells determined by flow cytometry is represented by green columns and that of the dead cells colored with propidium iodide (PI)—in red columns. As is revealed, the K2TS is the most efficient reagent for boosting the adenoviral transduction of MSC. Bars, 20 µm.

To increase the yield of the MSC transduction using low doses of adenovirus, we tested several potential boosters. For this, NOD-MSC were transduced with 250 TU/cell alone or in the presence of K2 Transfection System (K2TS), Lipofectamine 3000, polybrene, cholesterol, poly-L-Lysine, TransFast, or Viromer Red, as described in the Materials and Methods Section. Since the K2TS is currently used as a transfection reagent, we also used it for the transfection of MSC with the pAdTrack-CMV plasmid, which contains a GFP-encoding sequence. The results showed that from all the potential boosters tested, the K2TS was by far the most potent, as shown by fluorescence microscopy (Figure 2A). The transduction efficiency was quantified by flow cytometry. The data presented in Figure 2B (green columns) show that K2TS markedly increased the efficiency of the transduction by ~3.3 times (Figure 2B, AdV + K2 green column), whereas the other adjuvants (Lipofectamine 3000, polybrene, cholesterol, poly-L-Lysine, TransFast, and ViromerRed) did not increase the transduction yield (Figure 2B, green columns). After MSC transfection using K2TS (pAdTrack + K2), ~20% of the cells showed green fluorescence. The viability of the cells after transduction with or without other adjuvants was good; the dead cell percentages were between 3 and 10%, as detected following propidium iodide (PI) incorporation (Figure 2B, red columns). The complete data obtained by flow cytometry are shown in Supplemental Figure S1. Taken together, our result showed that the K2TS increased the number of transduced MSC and produced a robust expression of GFP as compared with AdV alone.

### 2.2. Optimization of the Adenoviral Transduction of MSC in the Presence of K2TS

K2TS comprises two components, K2 Multiplier (K2M), and K2 Transfection Regent (K2TR). To improve the yield of the adenoviral transduction, we determined the optimal dose of each component. For K2TS optimization we used 250 TU/mL adenovirus as this dose was the last point on the linear section of the dose-dependence curve. The yield of the transduction was recorded as the percentage of GFP-positive cells and the cell death was measured by PI incorporation in the cells, using flow cytometry. Since we obtained different transduction yields for MSC derived from distinct murine strains, we determined the effect of each component of the K2TS on each of the three strains-derived MSC.

#### 2.2.1. K2TR Optimization for Efficient Transduction Boosting

**Figure 3:**
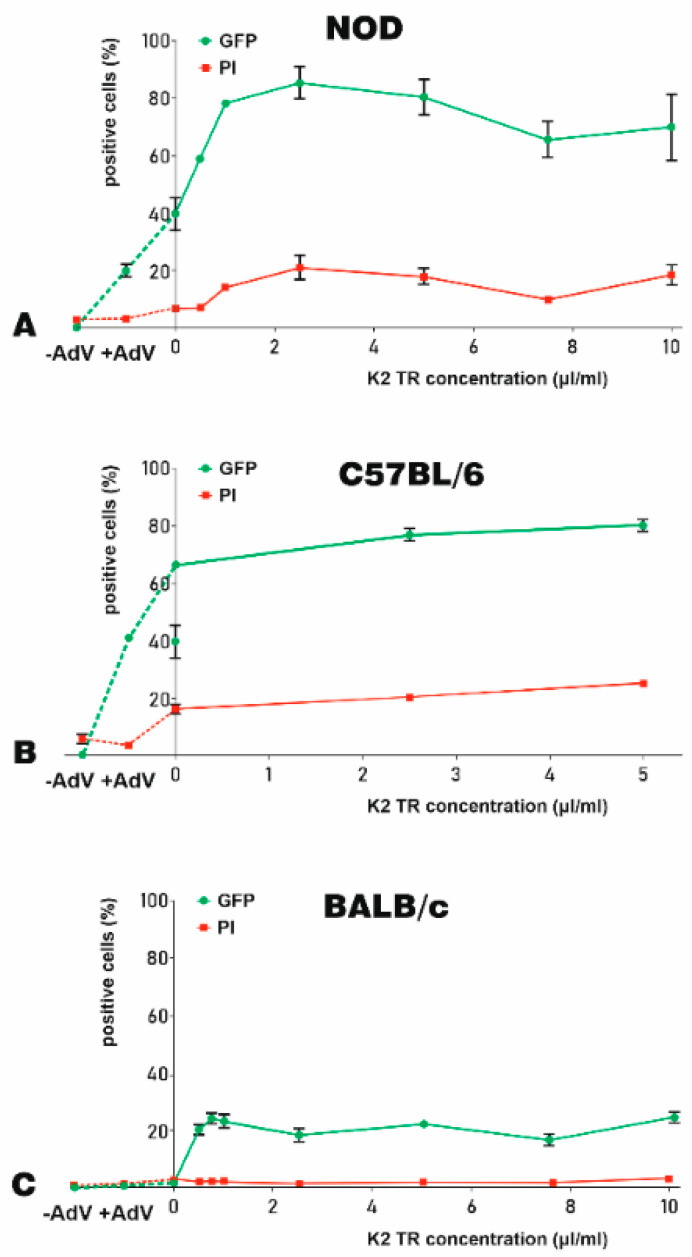
K2 Transfection Reagent K2TR optimization for the adenoviral transduction of murine MSC. The efficacy of K2TR to increase the yield of the adenoviral transduction of MSC derived from NOD (**A**), C57BL/6 (**B**), and BALB/c (**C**) mice were determined as % of GFP-positive cells (green lines). The cell death was determined by PI incorporation and expressed as % of PI-positive cells (red lines). On the left side of each graph, the GFP-positive cells and the cell death for the untransduced cells (−AdV) and for the cells transduced with 250 TU/cell adenovirus alone (+AdV) were illustrated, linked by a dotted line. Adenoviral particles were complexed with various K2TR doses. MSC were incubated with 10 μL/mL K2 Multiplier (K2M) and then the complexes of adenovirus–K2TR were added to the cells. The percentage of GFP-positive cells (green lines) induced by transduction and the percentage of PI-positive cells (red lines) were determined 48 h after transduction, by flow cytometry.

For K2TR optimization, the concentration of K2M was maintained constant. Thus, before transduction, 10^5^ NOD-, C57BL/6-, and BALB/c- MSC were incubated with 10 μL/mL of K2M for 90 min. Then, the cells were transduced with 5 × 10^7^ adenoviral particles (250 TU/cell) that were previously incubated for 20 min with 0.5–10 μL/mL of K2TR. Forty-eight hours after transduction, the cells were harvested and the percentage of GFP- and PI-positive cells were recorded by flow cytometry. To show a complete image of our results, we represent in Figure 3, on the left part of the graphs, the percentage of GFP-positive cells, as well as the cell-death for the untransduced cells (Figure 3, −AdV) and the cells transduced with the adenovirus alone (Figure 3, +AdV). The data showed that the K2TR augmented the yield of transduction, increasing the percentage of GFP-positive cells, but the shapes of the curves are different for the MSC derived from the three mice strains analyzed, as shown in Figure 3A–C.

NOD-MSC displayed a linear increase in the percentage of transduced, GFP-positive cells at doses lower than 2.5 μL/mL of K2TR. Note that the yield of transduction increased from ~20% in the absence of the booster (Figure 3A, −AdV), to 40% in the presence of K2M alone, without K2TR (Figure 3A). The maximum transduction yield (92%) was obtained for 2.5 μL/mL K2TR (in the presence of K2M). Higher K2TR concentrations did not further increase the transduction yield, but rather slightly decreased it to 79% for 10 μL/mL of K2TR (Figure 3A, green line). The cell-death ranged between 5 and 20% at different K2TR concentrations (Figure 3A, red line).

C57BL/6-MSC were more susceptible to adenoviral transduction; therefore, the adenovirus alone induced 40% GFP-positive cells, and the use of K2M in the absence of K2TR increased the transduction yield to ~70%, while the addition of K2TR raised it to 83%, as shown in Figure 3B (green line). The death of MSC after transduction was ~20% in the presence of various K2TR concentrations (Figure 3B, red line).

MSC isolated from the BALB/c strain were less prone to adenovirus infection since only ~1% of the cells were transduced with the adenovirus in the absence of the booster (Figure 3C, +AdV). K2M alone did not increase the transduction yield, but the addition of small doses of K2TR (0.5–1 μL/mL) increased the transduction yield to a maximum of 26%; higher doses did not further increase the number of GFP-positive cells, as shown in Figure 3C (green line). The viability of BALB/c-MSC was not affected by the adenoviral transduction in the presence or absence of the K2TS, when 1–3% dead cells (PI-positive MSC) were determined (Figure 3C, red line).

Taken together, the K2TR increased the adenoviral transduction yield of all three mouse strains derived-MSC tested without inducing a significant death of the cells. The optimal transduction of NOD- and C57BL/6-MSC was induced by a dose of 2.5 μL/mL K2TR and in the case of BALB/c-MSC, the adenoviral transduction was increased by 1 μL/mL K2TR (up to 21% GFP-positive cells).

#### 2.2.2. K2M Optimization

**Figure 4:**
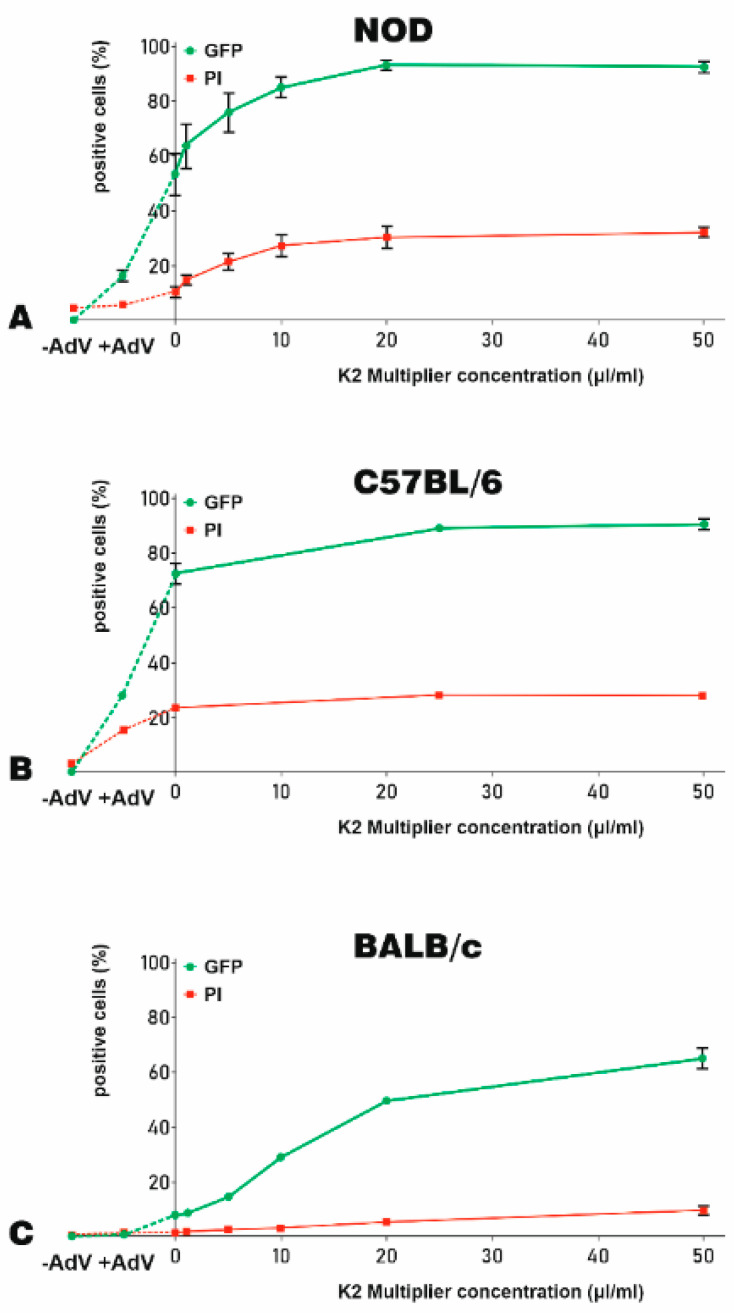
The optimization of K2M for adenoviral transduction of murine MSC. The efficacy of K2M to increase the adenoviral transduction of MSC-derived from NOD (**A**), C57BL/6 (**B**), and BALB/c (**C**) mice were expressed as % of GFP-positive cells (green lines). The K2M induced cytotoxicity was indicated by the % of PI-positive, dead cells (red lines). To optimize the concentration of K2M needed for transduction, MSC were incubated with increasing K2M concentrations (up to 50 μL/mL) for 90 min. Then, the adenovirus–K2TR complexes (5 μL/mL K2TR and 250 TU/cell adenoviral particle) were added to the cells. The percentage of GFP-positive cells and PI-positive cells at 48 h after transduction was determined by flow cytometry. The same fractions were evidenced also for untransduced cells (−AdV) and NOD-, C57BL/6- and BALB/c-MSC transduced with the adenovirus alone (+AdV), which were linked by a dotted line in the left side of the graphs.

Then, we studied the influence of K2M on the yield of adenoviral transduction of MSC isolated from NOD, C57BL/6, and BALB/c mice. For this, MSC were incubated for 90 min before transduction with different concentrations of K2M ranging from 1 to 50 μL/mL. The adenovirus (250 TU/cell) was complexed with 5 μL/mL K2TR and incubated overnight with the cells. The transfection yield and the death of the cells were evaluated 48 h later by flow cytometry. We determined the GFP and PI fluorescence for untransduced MSC (−AdV) or MSC transduced with the adenovirus alone (+AdV) as well as for cells treated with various amounts of K2M and transduced with adenoviral particles complexed with K2TR (Figure 4).

For NOD-MSC, K2M induced an increased GFP expression (up to 96%) when MSC were incubated before transduction with 20 μL/mL or 50 μL/mL K2M (Figure 4A, green line), as compared to 18% GFP-positive cells when adenovirus alone was used for transduction (Figure 4A, +AdV). However, increasing doses of K2M induced cell death, from 4% in the case of MSC transduced with adenovirus alone (Figure 4A, +AdV) to up to 35% when a concentration of 20 μL/mL or 50 μL/mL K2M was used to enhance the transduction (Figure 4A, red line).

The transduction yield of C57BL/6-MSC increased almost three-fold when K2M was used, reaching 86% GFP-positive cells (Figure 4B, green line), as compared with the adenovirus alone, when only 30% positive cells were detected (Figure 4B, +AdV). K2M did not have a negative impact on cell viability since the percentage of dead cells was similar in the absence of K2M to that recorded in the presence of different K2M concentrations (~22%, Figure 4B, red line).

BALB/c-MSC were less prone to adenovirus uptake, but the use of K2M greatly boosted the transduction yield. As shown in Figure 4C, the percentage of GFP-positive MSC increased from 0.6% obtained when the transduction was done with the adenovirus alone (+AdV) to 8% when the transduction was done in the presence 5 μL/mL K2TR and in the absence of K2M, reaching a maximum of 63% when 50 μL/mL K2M and 5 μL/mL K2TR were used (Figure 4C, green line). The mortality of BALB/c-MSC increased to a maximum of 8% reached after exposure to 50 μL/mL K2M, as compared with only 1% after transduction with the adenovirus alone (Figure 4C, red line).

Taken together, our results showed that K2M increased the adenoviral transduction efficiency for MSC-derived from NOD, C57BL/6, or BALB/c mice.

#### 2.2.3. The Expression of Adenoviral Receptors in MSC Derived from Different Mouse Strains

**Figure 5:**
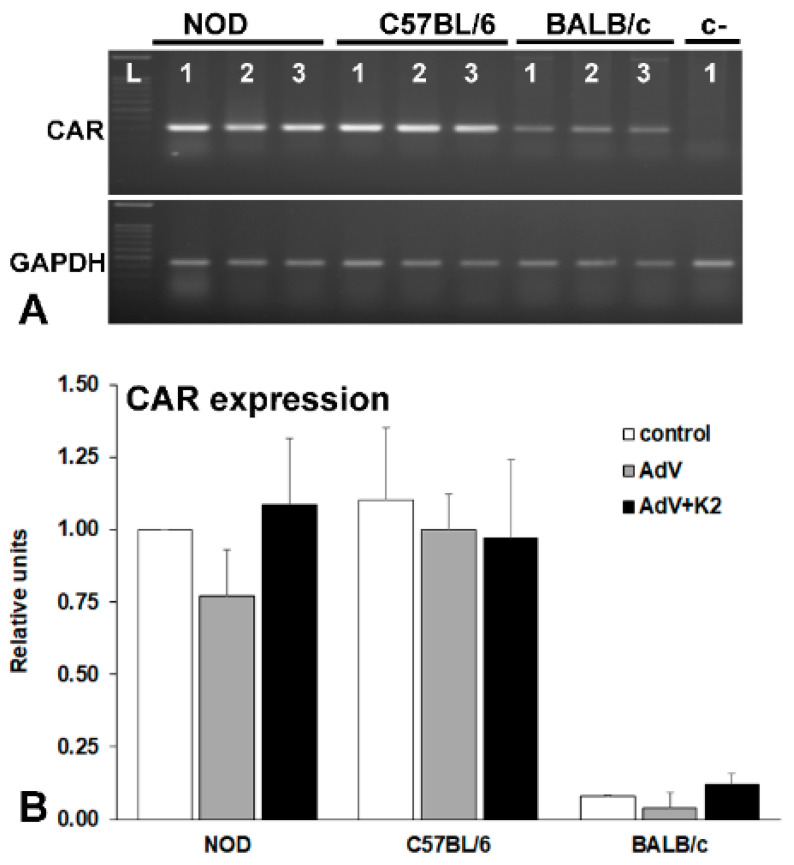
Evaluation of coxsackievirus and adenovirus receptor (CAR) expression in MSC derived from NOD, C57BL/6, and BALB/c mice analyzed by RT-PCR (**A**) and by Q-PCR (**B**). CAR expression is higher in NOD- and C57BL/6-MSC, as compared with that in BALB/c-MSC (**A**, lane 1 and **B**, white columns) and is not modified by the adenovirus (**A**, lane 2 and **B**, grey columns) or by the K2TS (**A**, lane 3 and **B**. black columns).

To evaluate the expression of the coxsackievirus and adenovirus receptor (CAR) in MSC derived from different murine strains, and to determine if the receptor is upregulated by the adenovirus in the presence or absence of the K2TS, we performed RT-PCR experiments (Figure 5A) and the relative level of CAR expression was quantified by Q-PCR (Figure 5B). The results showed that MSC from NOD and C57BL/6 mice strongly expressed CAR while in BALB/c-MSC the expression was very low (~5% of the level of expression obtained in NOD- or C57BL/6-MSC), as illustrated in Figure 5A, lane 1 and Figure 5B, control—white columns.

The adenovirus alone (Figure 5A, lanes 2, and Figure 5B, AdV—grey columns) or the presence of K2TS (Figure 5A, lanes 3 and Figure 5B, AdV + K2—black columns) did not influence the level of CAR expression.

### 2.3. Evaluation of the MSC Properties Following Adenoviral Transduction in the Presence of K2TS

To determine if the transduction conditions affect the characteristics of MSC, we checked the expression of some specific markers and assessed the differentiation potential and the immunosuppression capacity of the adenovirus transduced cells.

#### 2.3.1. Exposure to K2TS Does Not Modify the Expression of MSC Markers

**Figure 6:**
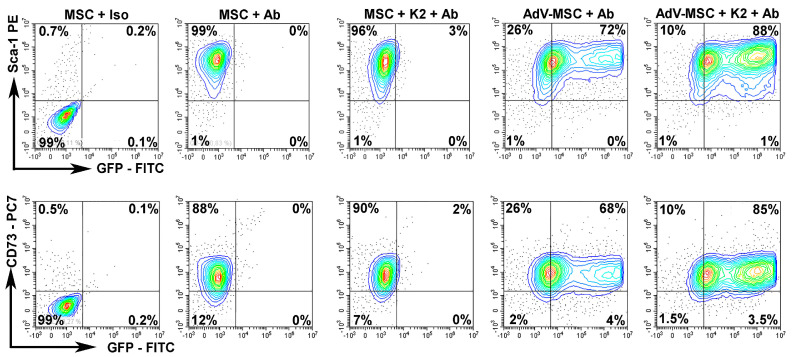
K2TS does not alter the markers expressed by MSC. The expression of Stem cells antigen-1 (Sca-1) and 5′-nucleotidase (CD73) was tested in NOD-MSC by flow cytometry. MSC were incubated with the corresponding isotype antibody (MSC + Iso) to set the gates. MSC were incubated with the corresponding antibodies (MSC + Ab) or were treated with K2TS and incubated with the antibodies (MSC + K2 + Ab). In parallel, MSC were transduced with the adenovirus alone (AdV-MSC + Ab) or in the presence of K2TS (AdV-MSC + K2 + Ab). In the upper panel the specific antibodies were anti-Sca-1 and in the lower panel were anti-CD73 antibodies. Both transduced and untransduced cells expressed a high amount of the markers (~98%). The GFP expression was detected in 16–20% of the cells transduced with the adenovirus alone (AdV-MSC + Ab) and in 90 - 92% in cells transduced with the adenovirus together with the K2TS (AdV-MSC + K2 + Ab).

First, we evaluated if the transduction conditions affect the expression of the MSC-specific markers. We analyzed the expression of Sca-1 and CD73 by flow cytometry in untransduced cells and in cells transduced in the presence or absence of the K2TS. Our data showed that Sca-1 (Figure 6, upper panels) and CD73 (Figure 6, lower panels) were unaffected by the transduction, being expressed at similar levels (~98% for each of the two markers) by the untransduced cells (Figure 6, MSC + Ab) or by the cells transduced using the adenovirus alone (Figure 6, AdV-MSC + Ab) or together with the K2TS (Figure 6, AdV-MSC + K2 + Ab). 

#### 2.3.2. Exposure to K2TS Does Not Influence the Multipotency of MSC

**Figure 7:**
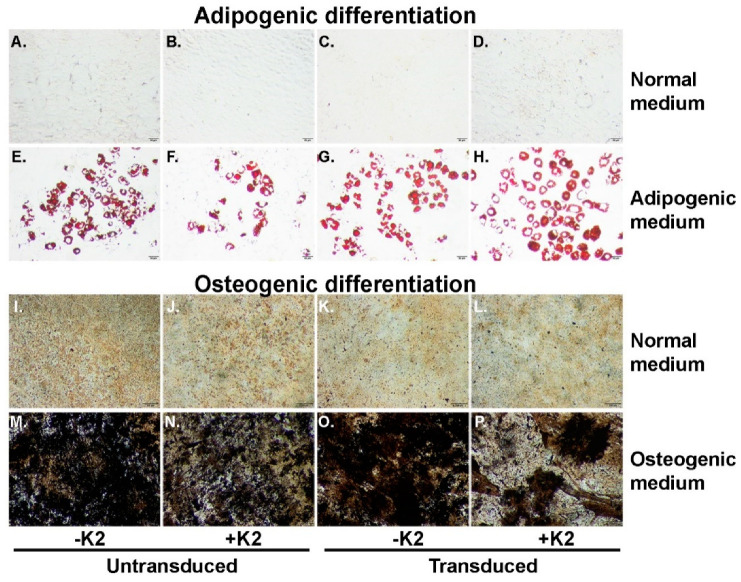
Exposure to K2TS does not influence the multipotency of MSC. Unmanipulated cells (**A**,**E**,**I**,**M**), K2TS-treated, untransduced (**B**,**F**,**J**,**N**), AdV-transduced (**C**,**G**,**K**,**O**), and AdV-transduced in the presence of K2TS (**D**,**H**,**L**,**P**) were cultured in normal MSC medium (**A**–**D**) and (**I**–**L**), or in adipogenic medium (**E**–**H**) or in osteogenic medium (**M**–**P**) for two weeks. The cells treated as described above were stained with Oil Red O (**A**–**H**), or with von Kossa protocol (**I**–**P**). Bars are 50 µm (**A**–**H**) and 200 µm (**I**–**P**).

Second, to determine whether K2TS exposure has an impact on the properties of MSC, we induced the adipogenic and osteogenic differentiation of (i) naïve cells, (ii) cells treated with K2TS, and cells transduced in the presence (iii) or absence (iv) of K2TS. As shown in Figure 7, NOD-MSC cultured in normal medium for 14 days did not display Oil Red O (Figure 7A–D) or von Kossa staining (Figure 7I–L), regardless of exposure to K2TS, or transduction in the presence or absence of K2TS. Moreover, after incubation with the adipogenic medium, naïve MSC showed robust differentiation into adipocytes as revealed by Oil Red O staining (Figure 7E). The transduction of MSC with AdV did not influence their adipogenic differentiation capacity (Figure 7F). Similarly, the treatment of the cells with K2TS alone, or their transduction with AdV in the presence of K2TS did not impact the adipogenic potential of MSC, as shown in Figure 7G,H. Exposing MSC to the osteogenic medium resulted, as expected, in the accumulation of calcium crystals confirmed by the von Kossa staining (Figure 7M). As observed in Figure 7N,O, neither the K2TS treatment nor the AdV transduction in the presence or absence of K2TS displayed any significant difference in the intensity of the von Kossa staining. Taken together, these results suggest that the K2TS did not influence the differentiation potential of MSC.

#### 2.3.3. The Immunosuppressive Properties of MSC Are Not Affected by the K2TS

Third, the immunomodulatory ability of K2TS-treated MSC was evaluated in a co-culture system using splenocytes from BALB/c mice. The splenocytes were labeled with carboxy-fluorescein succinimidyl ester (CFSE) and then activated with anti-CD3/anti-CD28 beads and incubated for 3 days onto monolayers of untreated MSC, MSC treated with the K2TS or transduced in the absence or presence of the K2TS. At the end of the incubation period, the splenocytes were harvested and the occurrence of T and B cell populations within the living cells was analyzed by flow cytometry. The gating strategy for stimulated splenocytes is shown in Supplemental Figure S2. The proliferation index (P.I.) was calculated based on the analysis of intracellular CFSE dilution determined for each of the CD45, CD4, and CD8 populations of splenocytes. In Supplemental Figure S3, an example is shown for the case of the CD8 T subset.

**Figure 8:**
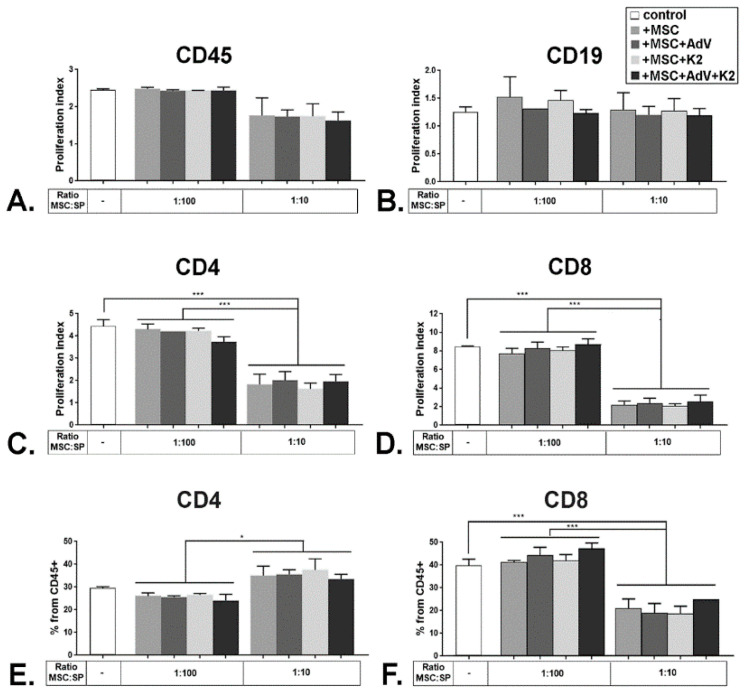
Immunomodulatory properties of MSC are not affected upon exposure to KTS. Whole splenocytes were labeled with carboxy-fluorescein succinimidyl ester (CFSE) and cultured in the presence of CD3/CD28 stimulation beads for 72 h alone (control), or together with naïve MSC (+MSC), transduced MSC (+MSC + AdV), K2TS-treated MSC (+MSC + K2) and transduced MSC in the presence of K2TS (+MSC + AdV + K2), in different ratios, as indicated. At the end of the incubation time, the proliferation index of the populations of splenocytes positive for CD45 (**A**), CD19 (**B**), CD4 (**C**), and CD8 (**D**) was calculated. The percentage of CD4+ (**E**) and CD8+ (**F**) cells from the CD45 positive splenocytes were determined and represented. * corresponds to a *p*-value < 0.05, and *** for *p* < 0.0005, by ANOVA.

As expected, splenocytes grown in co-culture with naïve MSC, displayed a near-total inhibition of proliferation for some sub-populations, when the ratio between MSC and splenocytes was 1:10. However, this inhibition was abrogated at a higher ratio, e.g., 1:100 (Figure 8). For the CD45^+^ population, the decrease was moderate, from a P.I. of 2.5 to 1.8, and did not reach statistical significance (Figure 8A, control vs. +MSC), probably because within the CD45 positive population there were cells that were not activated by the CD3/CD28 cue signal, such as the B cells (marked here by CD19), and thus they did not proliferate as much. The B cells reached a P.I. of 1.3 without MSC that was not changed in the presence of MSC, irrespective of the ratio between MSC and splenocytes (Figure 8B, control vs. +MSC). However, the proliferation of the CD4+ population was significantly inhibited from a P.I. of ~4.5 to 1.8, in the presence of naïve MSC, for a ratio of 1:10 MSC to splenocytes (Figure 8C, control vs. +MSC). The most dramatic proliferation inhibition was observed for the CD8+ cell population, when the P.I. decreased from 8.5 when the splenocytes were cultured alone, to ~2, when the ratio was 1:10 MSC to splenocytes (Figure 8D, control vs. +MSC). When mixed lymphocyte reaction experiments were performed co-incubating splenocytes with MSC transduced with the adenovirus alone (MSC + AdV), MSC treated with K2TS (MSC + K2) and MSC transduced in the presence of K2TS (MSC + AdV + K2), no significant differences in the immunomodulatory effect of MSC were observed (Figure 8A–D).

An important issue is how the proliferation inhibition affects the size of the CD4+ and CD8+ populations. To address this, we evaluated the percentage of CD4 and CD8 fractions within the CD45+ cells. We found that co-culturing of MSC with splenocytes at a ratio of 1:100 does not have any impact on both populations studied. When we used a ratio of 1:10 MSC to splenocytes, the CD8 population decreased significantly, from 38% to 20%, while the CD4 population increased from ~30% to 35% (Figure 8E,F). Thus, the strong inhibition of the proliferation of CD8 cells is reflected in the sharp decrease in the size of this population. However, even though the CD4 population displayed significant inhibition of proliferation, it showed a minor increase in size. This could be due to the occurrence of a subpopulation of T cells that were previously shown to be induced by the MSC, namely the T regulatory cells (Tregs, CD4 + CD25 + Foxp3+) [33]. All these results were recapitulated by the MSC that were exposed to K2TS in the presence or absence of AdV, as well as by the cells that were transduced with the AdV alone (Figure 8E,F). Considered together, these data demonstrate that K2TS does not change the immunomodulatory properties of MSC. Moreover, following the adenoviral transduction in the presence of K2TS, these characteristics are preserved in MSC.

In conclusion, our data recommend the K2TS as a great tool to ensure a more robust and reproducible transduction efficiency, coupled with a smaller adenovirus amount used to obtain a significant expression of exogenous proteins in hard to transduce, primary cells, such as the MSC.

## 3. Discussion

Many studies have demonstrated that MSC present low immunogenicity and have the potential to escape from the immune response of the host, being considered immune-privileged [34,35,36]. This feature enabled their administration across major histocompatibility barriers. However, some specific microenvironmental conditions such as hypoxia and differentiation lead to the loss of the immune privilege of allogeneic MSC [37]. An increasing number of data showed that allogeneic MSC produced an immune response and can be rejected by the recipient which reduces their therapeutic potential [38,39,40,41,42]. Thus, for some specific transplant experiments, syngeneic or autologous MSC are preferred. For example, NOD mice which represent a valuable tool for autoimmune diabetes have the H2g7 major histocompatibility complex, and thus, for syngeneic transplant, MSC should be derived from NOD mice not from C57BL/6 or BALB/c, which present a histocompatibility mismatch—H2kb to H2kd, respectively. By contrast, for studies related to Graft Versus Host Disease, the histocompatibility mismatch is mandatory and, thus, MSC from a proper mice strain will be used.

Here, we studied the transduction susceptibility of the MSC isolated from three mouse strains and we found major differences between the transduction yields obtained for each of them. For this, we used the adenoviral system AdEasy developed by B. Vogelstein [31] to pack an adenovirus carrying cDNA for GFP expression (AdV), as we previously described [32]. For titration, we employed a method based on flow cytometry that we previously described in detail [43]. This method was compared by Hit et al., 2000 [44] with the traditional plaque assay (to evaluate plaque-forming units, pfu), and they found that the results of the two methods gave similar results. The titration method based on flow cytometry that we used for the determination of the adenoviral titer is faster than plaque assay, reliable, and efficient. Moreover, in experiments in which we used 250 transduction units/cell (TU/cell) for C57BL/6-derived MSC we obtained similar transduction yields with those from the literature when 250–500 MOI/cell were used [45].

When high adenoviral doses were used (250 transduction units per cell), MSC from NOD and C57BL/6 mice, presented similar rates of transduction, while BALB/c-derived MSC were almost non-transduced, as detected by fluorescence microscopy (Figure 1). The very low transduction yield obtained for BALB/c-MSC were reported also by other researchers [26] who used polyethyleneimine (PEI) to facilitate the adenovirus uptake by the cells. For doses smaller than 100 TU/cell, NOD-MSC behaved differently in transduction as compared with C57BL/6-MSC. To our knowledge, there are no data in the literature showing adenoviral transduction of NOD-MSC. For low adenoviral doses, we observed a big difference between NOD-MSC and C57BL/6-MSC, the latter being more easily transduced.

Since a high number of transduced cells is needed for cell transplant and the adenoviral technology is expensive and time-consuming, we used some potential boosters shown in the literature to increase the yield of adenoviral transduction. Data from the literature revealed that poly-L-Lysine enhanced the transduction of MSC isolated from C57BL/6 mice [25] and PEI facilitated the adenovirus uptake by BALB/c-MSC [26]; other transduction boosters, such as Lipofectamine, increased the adenoviral transduction of hematopoietic cells [28], cholesterol facilitated the transduction of the human fibroblasts [29], and polybrene increased the transduction yield of mouse proteoblast progenitors [27]. However, in our hands, transduction-boosting molecules such as polybrene, Lipofectamine, free cholesterol, and poly-l-lysine were inefficient in NOD-MSC (Figure 2). Furthermore, we could not achieve an increase in the transduction yields by adding TransFast (containing a combination of synthetic cationic lipid, (+)-N,N [bis (2-hydroxyethyl)]-N-methyl-N- [2,3-di(tetradecanoyloxy)propyl]ammonium iodide and the neutral lipid, DOPE) or ViromerRed (containing alkylated and carboxyalkylated branched PEI). By contrast, the K2TS strongly boosted the transduction yield of MSC (Figure 2). This transfection system was previously shown to increase the yield of the gene electrotransfer in the B16F10 murine melanoma cell line [30]. K2TS has never been tested before as a booster for adenoviral transduction. The K2TS is a patented system (US20110045001A1) that contains two components K2M and K2TR. K2M is a cocktail of innate immune system inhibitors and antagonists that desensitizes the cells to foreign DNA by inhibiting several different factors involved in the signaling cascade after DNA detection, a mechanism described in [30]. Cell priming with K2M was very efficient and greatly enhanced the transduction (Figure 3). In the literature, it was reported that human MSC priming with 90–360 nM dexamethasone, a pharmacologic anti-inflammatory agent, increased the transfection efficiency [46]. The second component of K2TS, the K2TR is represented by cationic lipids which can form liposomes with foreign DNA. We demonstrated that small amounts of K2TR greatly facilitated the adenoviral transduction of MSC. We optimized the doses for K2M and K2TR to obtain the maximum MSC transduction yield. We observed that K2TS components enhanced the adenoviral transduction yield of MSC from NOD and C57BL/6 in a dose-dependent manner until they reached a plateau. For NOD- and C57BL/6-MSC, each K2TS component almost doubled the number of transduced cells; however, the K2M effect seemed to be slightly more pronounced than that of K2TR (Figure 3A,B and Figure 4A,B). For BALB/c-MSC, the effect of K2TR was more pronounced, and the optimal doses of both components raised the transduction yield from 1% to 64% (Figure 3C and Figure 4C).

The adenovirus attachment to the cell membrane is dependent on the CAR expressed by the target cells. To check if the mechanism of the transduction boosting in MSC is related to CAR upregulation, we assessed the CAR expression in MSC derived from NOD, C57BL/6, and BALB/c mice, in the presence of the K2TS (Figure 5). We observed that the CAR expression was not modified by the transduction and was not influenced by the K2TS components. We found a relatively strong CAR expression on MSC derived from NOD and C57BL/6, but a very low level of expression on BALB/c-MSC. This finding was in agreement with other reports regarding the low transduction efficiency of BALB/c-derived MSC (in the absence of the K2TS) [26]. All these data demonstrated that the transduction-boosting mechanism of K2TS is due to the capacity of K2M to desensitize the cells to the adenoviral DNA and to the ability of K2TR to facilitate the entry of the adenovirus into the MSC bypassing the CAR-mediated endocytosis. Commercially available CAR boosters increase CAR expression on the cell surface, which may be efficient for MSC derived from BALB/c mice. For NOD-derived MSC which already express a good amount of the receptors, this mechanism may not be required.

Genetic manipulation for therapeutic purposes should not alter the intrinsic properties of MSC. NOD-MSC used in our experiments expressed specific markers such as Sca-1, and CD73 (Figure 6). Here, we demonstrated that K2TS exposure did not affect the expression of Sca-1 and CD73 MSC markers (Figure 6). In addition, MSC transduced in the presence of K2TS preserved their potential to differentiate into the adipogenic and osteogenic lineages (Figure 7). Marasini et al. showed that the high doses of adenovirus needed to achieve efficient transduction of human MSC diminished their adipogenic capacity, but their potential to differentiate into chondrocytes and osteocytes was still maintained [47]. These data better emphasize the importance of the transduction boosting by K2TS, as it allows the use of lower adenoviral doses to attain a good expression of the transgene.

Another MSC feature relevant for therapy is represented by their immunosuppressive potential, as reviewed in [17,48,49]. In mixed cell cultures of transduced MSC together with allogeneic splenocytes, the cells directly interact through cell-to-cell contact, but also communicate through signaling molecules such as cytokines, including transforming growth factor-β (TGF-β), hepatocyte growth factor, and prostaglandin E2, as well as other anti-inflammatory factors [50,51]. It has been already established that activated T cells secrete a plethora of pro-inflammatory cytokines, including TNFα and IFNβ that induce apoptosis of MSC [52]. In our MSC-splenocytes co-cultures, MSC were exposed to this pro-inflammatory environment. However, following K2TS exposure, as well as after adenoviral transduction in the presence of K2TS, the immunomodulatory properties of MSC did not change (Figure 8), and the cells efficiently inhibited the proliferation of both CD4 and CD8 T cells. Our data suggest that the K2TS does not interfere with the pathways responsible for the immunosuppressive properties of MSC, like secretion of TGF-β, hepatocyte growth factor, iNOS [53,54].

In conclusion, K2TS proved to enhance the adenoviral transduction of MSC inducing an increased transgene expression, without affecting their intrinsic characteristics. This highly efficient method saves a lot of work, time, and money otherwise needed to obtain huge amounts of adenoviral particles for MSC transduction in the absence of a K2TS booster.

## 4. Materials and Methods

All reagents and chemicals were purchased from Sigma-Aldrich unless stated otherwise. DMEM with 1 g/l glucose and FBS-qualified MSC were purchased from PAN Biotech (PAN-Biotech GmbH, Aidenbach, Germany), FavorPrep™ Tri-RNA Reagent was from Favorgen (Favorgen Biotech Corp, Vienna, Austria), PacI Fast Digest enzyme, SYBR™ Green master mix, Dynabeads mouse T-Activator CD3/CD28 and Lipofectamine 3000 were from Thermo Fisher (Thermo Fisher Scientific, Waltham, MA, USA). Antibodies, anti-mouse Sca-1(PE), CD73(Cy7), CD45(PE), CD4(BV421), CD8a(APCfire), and CD19(APC) were from BioLegend (San Diego, CA, USA), and CFSE (5(6)-Carboxyfluorescein diacetate N-succinimidyl ester) were from Invitrogen (Fisher Scientific, part of Thermo Fisher Scientific, Waltham, MA, USA). Polybrene was purchased from Santa Cruz Biotechnology (sc 134220; Dallas, TX, USA), TransFast was from Promega (E2431, WI, USA), Viromer Red was from Cambridge Bioscience (VR-01LB-00, Cambridge, UK), the K2 Transfection System—DNA & RNA Transfection was from Biontex Laboratories GmbH (München, Germany).

### 4.1. MSC Isolation and Culture

C57BL/6 (Stock No: 000664), NOD/ShiLtJ (NOD; Stock No: 001976), and BALB/c (Stock No: 000651) mice were purchased from The Jackson Laboratory (Bar Harbor, ME, USA). The 8 weeks-old mice were killed by cervical dislocation, and the iliac bones, the femurs, and tibiae were harvested. The procedure was performed in accordance with the EU Directive 2010/63 and national laws (authorization no. 296/23.08.2016). The bone marrow was extracted by flushing the bones with cold DMEM with 1 g/L glucose supplemented with 10% MSC-qualified FBS (MSC medium) in a syringe with a 26-gauge needle, as previously described [55]. The single-cell suspension was obtained by gently passing the bone marrow through an 18-gauge needle, followed by filtration through a 40 µm nylon cell strainer. The cells were plated starting with a cell density of 2 × 10^6^ cells/cm^2^ in MSC medium with 1% Penicillin/Streptomycin/Amphotericin. Confluent cells were subjected to several passages, usually following a split of 1:3 to 1:4. For experiments, cells were seeded at 5000 cells/cm^2^. The cells used in experiments were characterized by MSC markers and by lineage differentiation. The cells in this study were used between passage 7 and 10.

### 4.2. Adenovirus Packaging, Purification, and Titration

The adenovirus was obtained and titrated as we previously described in detail [32,43]. Briefly, pAdTrack adenoviral vector containing DNA encoding GFP under CMV promoter (plasmid #16405, Addgene, Watertown, MA, USA) was linearized with PmeI and used to transform AdEasier-1 cells (BJ5183-bacteria containing the pAdEasy1 plasmid), a gift from Bert Vogelstein (#16399, Addgene Watertown, MA, USA). After selection by PCR and by digestion with the restriction enzymes, the recombinant DNA was amplified in DH5α. Then, the recombined plasmid was purified and was used for the transfection of AD293 cells, using the K2 transfection reagent (Biontex Laboratories GmbH, München, Germany). The adenovirus was further amplified in AD293 cells and then the virus was purified by ultracentrifugation at 35,000 rpm on a discontinuous CsCl gradient (1.2 g/L and 1.4 g/L), for 18 h at 4 °C.

To determine the titer, AD293 cells seeded in 12-wells plates were transduced with adenoviral dilutions ranging between 1/10^4^ and 1/10^7^. GFP-expressing cells were determined by flow cytometry and the transduction units were calculated using the formula. The samples with 5–20% GFP-positive cells from the total population were taken into account for the calculation of viral titer using the following formula: Titer (TU/mL) = D × F/100 × C/V, where D is the dilution factor, F is the percentage of positive cells/100, C is the counted cells/well, and V is the volume of viral inoculum.

### 4.3. Adenoviral Transduction of MSC

The adenoviral transduction experiments used MSC (isolated from NOD, BALB/c, and C57BL/6 mice) seeded in 6-well plates at a density of 1 × 10^5^ cells/well in 1 mL of normal MSC media, one day before transduction. The dose-dependent curve for the transduction efficiency was determined by incubating MSC with adenoviral doses ranged between 50 and 2500 TU/cell in 1 mL medium. A total of 18 h later, the medium was replaced with 2 mL fresh medium and cells were kept for another 48 h till they were analyzed.

### 4.4. Adenoviral Transduction of MSC in the Presence of Various Potential Boosters

A total of 1 × 10^5^ MSC were transduced with 2.5 × 10^7^ adenoviral particles (corresponding to 250 TU/cell) alone (AdV) or in complexes with some polymers or cationic lipids, prepared as follows: (i) AdV + K2: 90 min before transduction, MSC were incubated with 10 µL K2M; in the meantime, 2.5 × 10^7^ adenoviral particles were diluted in 65 µL DMEM. This viral dilution was mixed with 5 µL K2TR diluted in 65 µL DMEM. The mixture was incubated for 20 min at room temperature and then was added dropwise to the MSC; (ii) AdV + Lipofectamine: a mixture containing 2.5 × 10^7^ adenoviral particles and 10 µL P3000 in 125 µL Opti-MEM was added over a dilution of 7.5 µL Lipofectamine Reagent in 125 µL Opti-MEM; the final mix was incubated for 20 min at RT and then was added dropwise to the MSC; (iii) AdV + Polybrene: 2.5 × 10^7^ adenoviral particles were added in 250 µL DMEM containing 2.5 µL solution 10 mg/mL polybrene; the mixture was added dropwise to MSC; (iv) AdV + Cholesterol: 2.5 µL solution containing 0.4 mg/mL cholesterol (in ethanol) were mixed with 2.5 × 10^7^ adenoviral particles diluted in 250 µL DMEM and incubated for 20 min at RT; then the mixture was added dropwise to the MSC; (v) AdV + Poly-L-Lysine: 2.5 µL solution containing 0.1 mg/mL poly-L-Lysine were mixed with 2.5 × 10^7^ adenoviral particles in 250 µL DMEM, and then the mixture was incubated with MSC in 1 mL serum free medium for 90 min; after this, 100 µL FBS was added to the cells; (vi) AdV + TransFast: 21 µL TransFast in 100 µL DMEM were mixed with 2.5 × 10^7^ adenoviral particles and incubated for 15 min at RT, then were added to the MSC; (vii) AdV + Viromer Red: 540 µL Viromer Buffer with 2.5 × 10^7^ adenoviral particles were mixed with 2.5 µL Viromer Red in 57.6 µL Viromer Buffer and incubated 15 min at RT, then the mixture was added to the cells.

Transfection of MSC with pAdTrack-CMV using K2TS was performed following the manufacturer’s instructions. Briefly, MSC were incubated with 10 µL K2M for 90 min; in parallel, 2.4 µg pAdTrack-CMV diluted in 65 µL DMEM were mixed with 5 µL K2 transfection reagent diluted in 65 µL DMEM and incubated 20 min at RT; then, the mixture was added dropwise to the MSC. Eighteen hours later, the medium was replaced and the cells were analyzed 48 h post-transduction. The presence of GFP was evaluated by fluorescence microscopy at 20× (Axio Vert A.1, Carl Zeiss Jena GmbH, Jena, Germany), or quantified by flow cytometry using CytoFLEX Flow Cytometer (Beckman Coulter Life Sciences, IN, USA).

### 4.5. Optimization of the K2TS Components for MSC Adenoviral Transduction

The doses of both K2TS components, K2TR and K2M were optimized. (i) To optimize K2TR concentration, MSC were incubated for 90 min with 10 μL/mL K2M in the culture medium. In parallel, 2.5 × 10^7^ adenoviral particles diluted in 65 μL DMEM were mixed with increasing doses of K2TR (0.5–10 μL) diluted in 65 μL DMEM and incubated for 20 min at room temperature. (ii) To optimize K2M concentration, MSC were incubated with 1–50 μL K2M for 90 min. The complexes of the adenoviral particles with 5 μL K2TR were done as described above. In both cases, the mixture was added drop by drop to the MSC. After 18 h the medium was changed and the GFP expressing cells were analyzed 48 h after transduction.

For the experiments of lineage differentiation, CAR detection, MSC markers expression, and immunomodulation, the procedure was similar as above, using 10 μL/mL K2M and 5 µl/mL K2TR.

### 4.6. RT-PCR and Quantitative Real-Time PCR

Transduced or untransduced MSC isolated from NOD, BALB/c, and C57Bl/6 mice were lysed in FavorPrep™ Tri-RNA Reagent, and total RNA was extracted according to the manufacturer’s instructions. cDNA was synthesized from 1 µg of RNA using oligo(dT) primers and M-MLV reverse transcriptase. Murine CAR expression was determined by PCR and quantified by Q-PCR using the specific primers (F5′-GCCATCCTCTTCTGCTGTCAC, R5′-GCAGGAATCATCACAGGAACCG) which generate products of 307 bp. GAPDH expression was also determined for normalization. PCR was performed using Go-Taq Polymerase (35 cycles for CAR and 30 cycles for GAPDH) using SensoQuest PCR (SensoQuest GmbH, Göttingen, Germany) and Q-PCR experiments were performed using SYBR™ Green master mix using a 7900 HT Applied Biosystems machine (Applied Biosystems, part of Thermo Fisher Scientific, Waltham, MA, USA).

### 4.7. MSC Differentiation

To evaluate the multipotency of MSC exposed to K2TS, cells were cultured at a 5000 cell/cm^2^ density in a 24-well plate and allowed to adhere overnight. Cells were treated with K2TS and transduced with AdV as described above. Twenty-four 24 h after transduction, media was changed with adipogenic and osteogenic induction media, respectively, as well as normal MSC media. Every 2–3 days, the media was refreshed. The adipogenic induction media contained DMEM containing 1g/L glucose supplemented with 10% MSC-FBS, 10^−6^ M dexamethasone, 100 mM indomethacin and 1% Insulin-Transferrin-Selenium (ITS-G, ThermoFisher, Waltham, MA, USA). The osteogenic induction media was based on DMEM with 1 g/L glucose supplemented with 10% MSC-FBS, 10^−7^ M dexamethasone, 10 mM β-glycerophosphate, 0.3 mM ascorbic acid. After 2 weeks, cells were fixed with 4% paraformaldehyde and stained with Oil Red O to visualize the lipid droplets accumulated in the differentiated adipocytes. Alternatively, after fixation, cells were labeled with the von Kossa stain by incubation in 5% AgNO_3_, followed by a short 2-min rinse with 5% sodium thiosulphate to visualize the calcium crystals. All the images were taken using an Olympus CKX41 microscope with an Olympus XC30 camera.

### 4.8. Immunosuppression Assay

To determine the immunomodulatory properties of MSC transduced with AdV with or without the K2TS, cells were plated in a 24 well plate at two different densities, 5 × 10^4^ and 5 × 10^3^. The next day, MSC were transduced as previously described. A total of 24 h later, the medium was removed, and 5 × 10^5^ freshly isolated whole splenocytes isolated from BALB/c mice and pre-labeled with 2.5 mM carboxy-fluorescein succinimidyl ester (CFSE) were added in RPMI supplemented with 10% FBS, 1% PSA, and 50 µM β-mercaptoethanol in the presence of CD3/CD28 stimulation beads (in a ratio of 1:1 beads to splenocytes). Two control experiments were performed: (i) unstimulated splenocytes were incubated with MSC, (ii) stimulated splenocytes incubated with beads, without MSC. The cells were incubated for 72 h, and then the splenocytes were collected by gently detaching them from the MSC with 0.05 mM EDTA in phosphate-buffered saline (PBS, 10 mM phosphate with 137 mM NaCl, pH 7.4), stained with antibodies for lymphocyte population-specific markers and analyzed by flow cytometry.

### 4.9. Flow Cytometry

The efficiency of MSC transduction was estimated as the percentage of the number of GFP-positive cells from the total number of the cells, and cell death was measured as the percentage of cells which incorporated propidium iodide (PI) from the total number of cells. Briefly, 48 h post-transduction, MSC were washed with PBS, detached with 0.125% trypsin/1 mM EDTA, pelleted by centrifugation at 400× *g*, 5 min, 4 °C, and were resuspended in 0.25 mL FACS buffer (PBS with 2% fetal bovine serum). Just before analysis, 1 μL PI (100 μg/mL stock) was added to each sample and run on the CytoFLEX machine (Beckman Coulter, Indianapolis, IN, USA).

To determine the MSC markers, 10^5^ MSC/sample were incubated with PBS with 2% fetal bovine serum and 5% mouse serum, then were stained with antibodies against Sca-1(PE) and CD73 (PE/Cy7) and run on CytoFLEX.

For the immunosuppression assay, the splenocytes were labeled with an antibody mixture containing anti-mouse CD45PE, anti-mouse CD4BV784, anti-mouse CD8aAPCfire, and anti-mouse CD19APC and run on the CytoFLEX (Beckman Coulter, Indianapolis, IN, USA) flow cytometer.

All the data recorded by CytoFLEX were analyzed with CytExpert flowing software. Cells were first gated using corresponding isotype antibodies. The proliferation index of the splenocytes was estimated based on the CFSE analysis, using the unstimulated splenocytes as the parent (basal fluorescence); the data were processed using ModFit LTTM software (Verity Software House Topsham, ME, USA).

### 4.10. Statistical Data Analysis

All the experiments were done in triplicate and were repeated three–four times. Analysis of the data was performed using the GraphPad Prism Software. Data are presented as ±SEM or SD, as is indicated in the text and the figure legends. A *p*-value < 0.05 was considered statistically significant.

## 5. Conclusions

Our data showed that K2TS boosts the adenoviral transduction of MSC. This system makes possible the induction of the transgene expression in MSC infected with low doses of adenovirus. The cell surface-specific markers, the pluripotency, and the immunomodulatory capacity of the MSC were unaffected when the transduction was performed in the presence of K2TS.

## Data Availability

Data are contained and available within this manuscript.

## Supplementary Materials

Supplementary Materials can be found at https://www.mdpi.com/1422-0067/22/2/598/s1. Figure S1. Flow cytometric data showing the GFP expression in murine mesenchymal stromal cells (MSC) transduced in the presence of different potential transduction boosters. Figure S2. Gating strategy for stimulated splenocytes. Figure S3. K2TS does not influence the immunomodulatory properties of MSC.

## Abbreviations

AdV	Adenovirus
CAR	Coxsackievirus and Adenovirus Receptors
CFSE	Carboxy-fluorescein succinimidyl ester
GFP	Green fluorescent protein
K2M	K2 Multiplier
K2TR	K2 Transfection Reagent
K2TS	K2 Transfection System
MOI	multiplicity of infection
MSC	Mesenchymal Stromal Cell
NOD	Non-Obese Diabetic
PI	Propidium iodide
Sca-1	Stem Cells Antigen-1
TU	Transduction Units
