# Supplementary material for: K2 Transfection System Boosts the Adenoviral Transduction of Murine Mesenchymal Stromal Cells
Source: Int J Mol Sci. 2021 Jan 9;22(2):598. doi: 10.3390/ijms22020598 (PMC7826527; doi:10.3390/ijms22020598)
Supplement: Supplementary file 1 [file ijms-22-00598-s001.pdf]

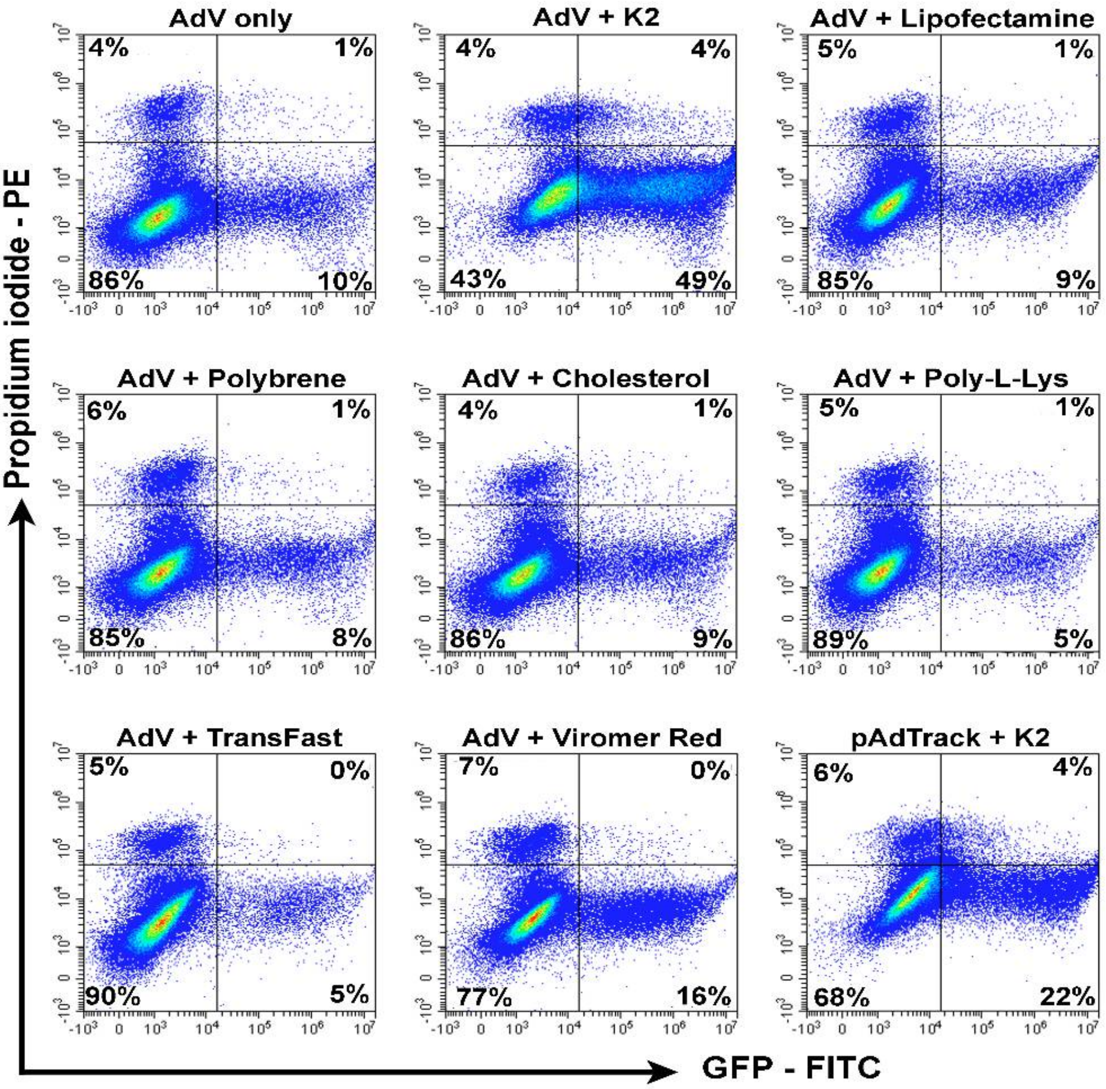

Figure S1. Flow cytometric data showing the GFP expression in murine mesenchymal stromal cells (MSC) transduced in the presence of different potential transduction boosters. To induce GFP expression, MSC were incubated with 250 TU /cell adenovirus alone (AdV only) or in the presence of K2TS (AdV+K2), Lipofectamine 3000 (AdV+Lipofectamine), 10 µg/ml Polybrene (AdV+Polybrene), 2µg/ml free cholesterol (AdV+Cholesterol), 1µg/ml poly-L-Lysine (AdV+Poly-L-Lys), TransFast (AdV+TransFast), or Viromer Red (AdV+ViromerRed). In parallel, MSC were transfected with pAdTrack-CMV using K2TS (pAdTrack+K2). After 48 hours, the GFP expression was determined by flow cytometry. The percentage of GFP-positive cells and the dead cells colored with propidium iodide (PI) were determined. As revealed, the K2TS is the most efficient reagent for boosting the adenoviral transduction of MSC. Here is presented one representative experiment, out of three experiments performed.

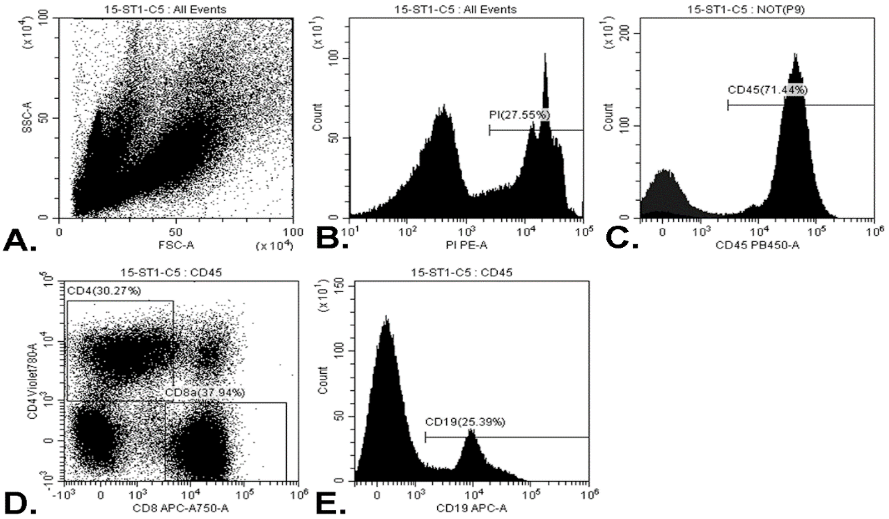

Figure S2. Gating strategy for stimulated splenocytes. (A) Splenocytes were cultured in the presence of CD3/CD28 stimulation beads for 72 hours and then evaluated by flow cytometry on SSC and FSC. (B) Cells were stained with PI and gated out to obtain the viable cells. (C) Cells that were negative for PI, were gated on CD45. Within the CD45+ cells, we gated on CD4+ and CD8+ cells (D) and CD19+ cells (E).

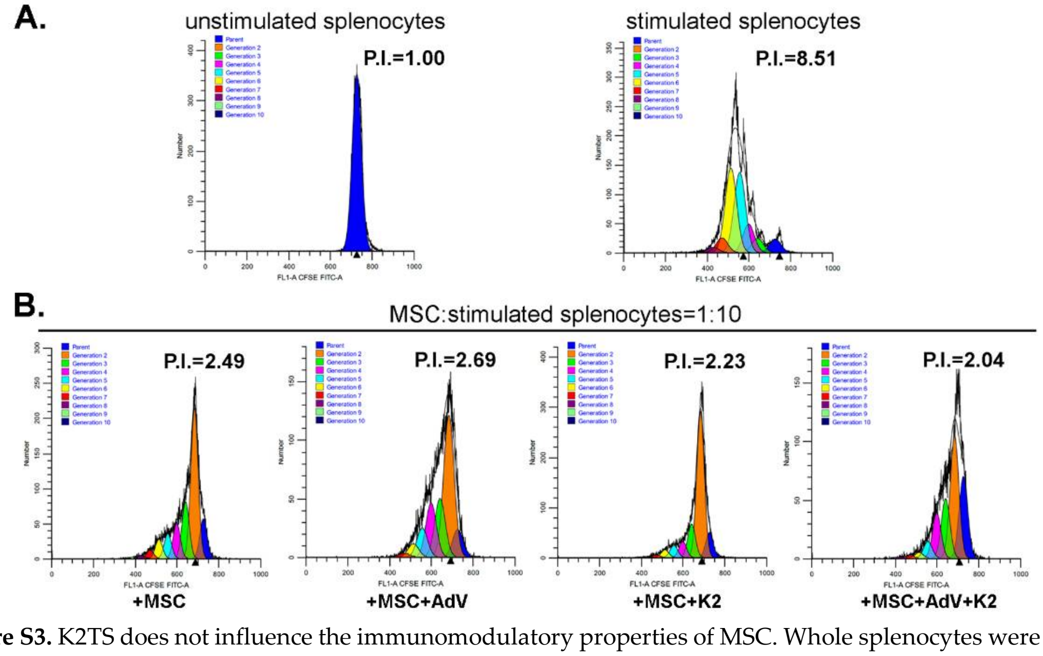

Figure S3. K2TS does not influence the immunomodulatory properties of MSC. Whole splenocytes were labeled with CFSE and cultured in the absence (unstimulated splenocytes) or presence (stimulated splenocytes) of CD3/CD28 stimulation beads for 72h alone (A), or together with naïve MSC (+MSC), transduced MSC (+MSC+AdV), K2TS-treated MSC (+MSC+K2) and transduced MSC in the presence of K2TS (+MSC+AdV+K2), in 1:10 ratios, as indicated (B). At the end of the incubation time, the proliferation index of the populations of CD45+CD8+ splenocytes was calculated using the ModFit software and indicated for each diagram.
